# Apocarotenoids: Old and New Mediators of the Arbuscular Mycorrhizal Symbiosis

**DOI:** 10.3389/fpls.2019.01186

**Published:** 2019-09-27

**Authors:** Valentina Fiorilli, Jian You Wang, Paola Bonfante, Luisa Lanfranco, Salim Al-Babili

**Affiliations:** ^1^Department of Life Sciences and Systems Biology, University of Torino, Torino, Italy; ^2^The BioActives Lab, Division of Biological and Environmental Science and Engineering, King Abdullah University of Science and Technology, Thuwal, Saudi Arabia

**Keywords:** carotenoids, apocarotenoids, strigolactones, abscisic acid, mycorradicin, blumenols, zaxinone, arbuscular mycorrhizal symbiosis

## Abstract

Plants utilize hormones and other small molecules to trigger and coordinate their growth and developmental processes, adapt and respond to environmental cues, and communicate with surrounding organisms. Some of these molecules originate from carotenoids that act as universal precursors of bioactive metabolites arising through oxidation of the carotenoid backbone. This metabolic conversion produces a large set of compounds known as apocarotenoids, which includes the plant hormones abscisic acid (ABA) and strigolactones (SLs) and different signaling molecules. An increasing body of evidence suggests a crucial role of previously identified and recently discovered carotenoid-derived metabolites in the communication with arbuscular mycorrhizal (AM) fungi and the establishment of the corresponding symbiosis, which is one of the most relevant plant–fungus mutualistic interactions in nature. In this review, we provide an update on the function of apocarotenoid hormones and regulatory metabolites in AM symbiosis, highlighting their effect on both partners.

## Introduction

Carotenoids are a group of lipophilic, isoprenoid pigments characterized by bright colors ranging from yellow to red. Plant carotenoids consist of a common C_40_ skeleton and differ with respect to the number and stereo-configuration of conjugated double bonds, the presence of oxygen, and structure of end groups (Hirschberg, 2001; Moise et al., 2014). These pigments are best known for their essential role in photosynthesis where they protect the photosynthetic apparatus from photooxidative damage, act as accessory pigments absorbing photons and transferring them to chlorophyll, and stabilize thylakoid membranes. In addition, carotenoids are frequently accumulated in flowers and fruits, serving as optical signal in plant–animal communication (Fraser and Bramley, 2004; DellaPenna and Pogson, 2006; Nisar et al., 2015). Besides these plant specific functions, carotenoids are the precursors for different biologically important compounds in all clades of life, which include retinoids, hormones, and signaling molecules (Nisar et al., 2015). These carotenoid-derivatives are formed by oxidative cleavage of their precursor, which produces a wide range of compounds generally called apocarotenoids (Giuliano et al., 2003; Nisar et al., 2015).

**Figure 1:**
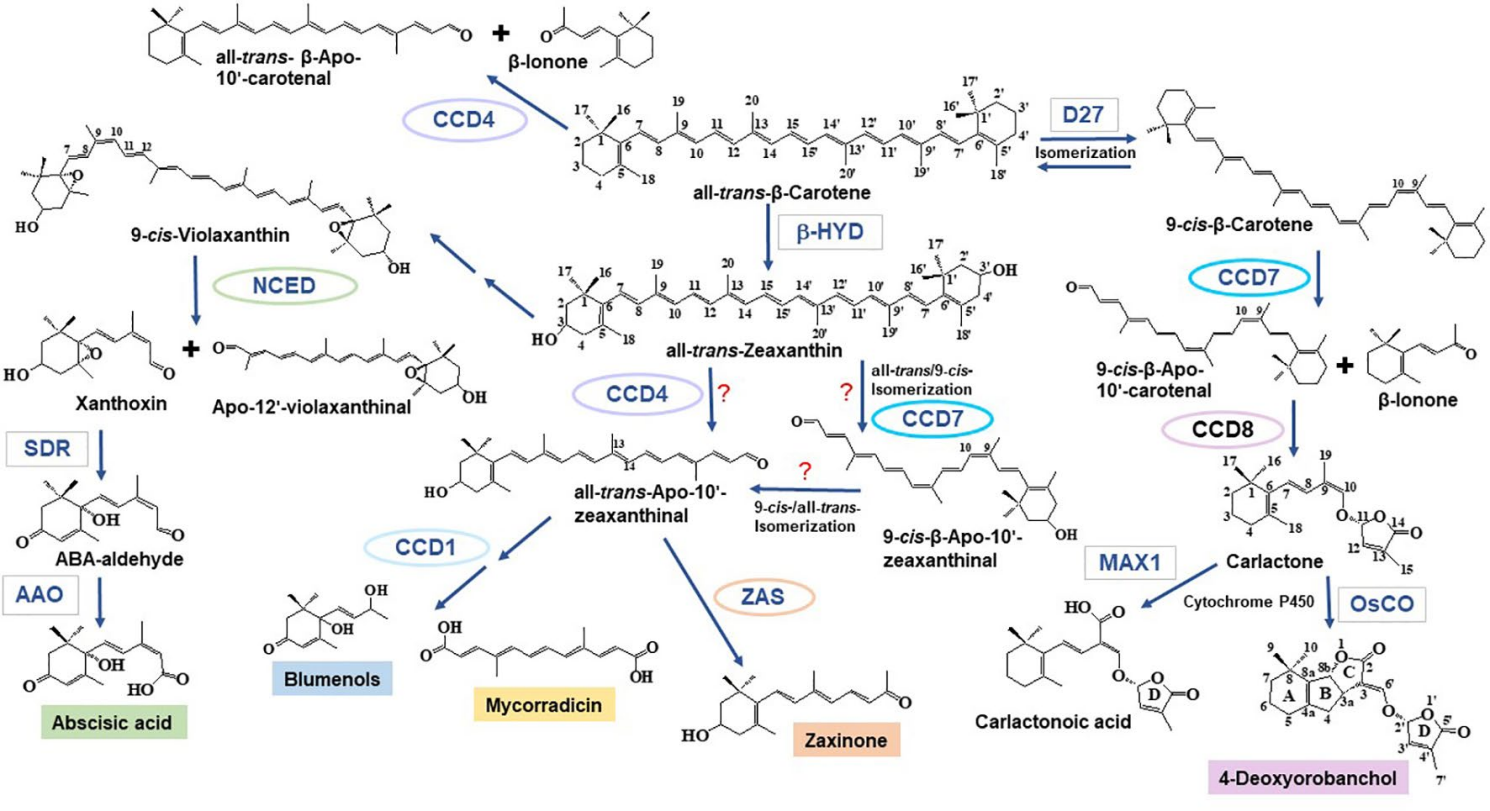
Formation of apocarotenoids involved in mycorrhization. Nine-*cis*-epoxycarotenoid dioxygenase (NCED) enzymes catalyze the cleavage of 9-*cis*-violaxanthin—formed from all-*trans*-zeaxanthin through epoxidation and isomerization reactions—and 9’-*cis*-neoxanthin (not shown) into the ABA precursor xanthoxin and apo-12’-violaxanthinal (or apo-12-neoxanthinal, not shown) (Nambara and Marion-Poll, 2005). Xanthoxin is then further converted to ABA by SDR and AAO. Carotenoid cleavage dioxygenase (CCD) enzymes catalyze a set of different carotenoid and apocarotenoid cleavage reactions. The C_27_ apocarotenoids β-apo-10’-carotenal and/or β-apo-10’-zeaxanthinal may be formed by CCD4 enzymes that cleave all-*trans*-bicyclic carotenoids (Bruno et al., 2015; Bruno et al., 2016). CCD7 has been also implicated in the formation of all-*trans*-β-apo-10’-carotenoids, which include the zaxinone precursor all-*trans*-β-apo-10’-zeaxanthinal (see below); however, in this case, a *cis* to *trans* isomerization must be postulated, as the apo-10’-carotenoids produced by CCD7 enzymes are 9-*cis*-configured (Alder et al., 2012; Bruno et al., 2014). Several enzymatic studies show that CCD1 enzymes can produce C_14_ directly from carotenoids or—in a secondary cleavage reaction—from all-*trans*-β-apo-10’-carotenoids. In the case of mycorrhizal tissues, it is assumed that they use β-apo-10’-carotenoids as substrate to form precursors of mycorradicin and blumenols (structure shown as blumenol C), which accumulate in AM-colonized root and act as symbiosis signal in plant leaves, respectively (Floss et al., 2008b; Walter et al., 2010; Hou et al., 2016; Wang et al., 2018). Following β-carotene isomerization catalyzed by D27, the SL biosynthetic enzyme, CCD7, cleaves 9-*cis*-β-carotene into 9-*cis*-β-apo-10’-carotenal and β-ionone. This step is followed by the CCD8-catalyzed conversion of 9-*cis*-β-apo-10’-carotenal into carlactone. Carlactone, a central intermediate in SL biosynthesis, is further modified by cytochrome P450 enzymes of the 711 clade (i.e., the Arabidopsis MAX1 (Abe et al., 2014), the rice carlactone oxidase (Zhang et al., 2014), which yield canonical, e.g., 4-deoxyorobanchol, and non-canonical, e.g., carlactonoic acid, SLs. Carlactonoic acid is further modified into different products (Alder et al., 2012; Bruno et al., 2014; Al-Babili and Bouwmeester, 2015; Bruno et al., 2017; Abuauf et al., 2018; Jia et al., 2018). ZAS, a recently identified CCD, cleaves apo-10’-zeaxanthinal, yielding the novel signaling molecule, zaxinone (Wang et al., 2019). β-Apo-10’-zeaxanthinal could be formed from zeaxanthin or lutein (not shown) by CCD4 enzymes. Enzymes are surrounded either by ellipses (CCDs) or rectangles (other enzymes). SDR, short chain dehydrogenase reductase; AAO, Abscisic aldehyde oxidase; β-HYD, β-hydroxylase; D27, DWARF27; MAX1, more axillary growth1; OsCO, rice carlactone oxidase, a MAX1 homolog.

Carotenoid cleavage can take place as a non-enzymatic process induced by reactive oxygen species that arise especially under stress conditions (Ramel et al., 2012; Havaux, 2014). However, the formation of most of the plant apocarotenoid hormones and signaling molecules involves carotenoid cleavage dioxygenases (CCDs), an evolutionarily conserved family of non-heme Fe^2+^-dependent enzymes, present in all taxa (Giuliano et al., 2003; Moise et al., 2005; Hou et al., 2016). A recent survey on plant CCDs identified six subfamilies: NCED (nine-*cis*-epoxycarotenoid dioxygenases), CCD1, CCD4, CCD7, CCD8, and zaxinone synthase (ZAS) (Wang et al., 2019). In brief, NCEDs catalyze the first step in abscisic acid (ABA) biosynthesis by cleaving 9-*cis*-violaxanthin or 9’-*cis*-neoxanthin at the C11, C12 or C11’, C12’ double bond, respectively, forming the ABA precursor xanthoxin, which is further metabolized by short chain dehydrogenase reductase (SDR) and abscisic aldehyde oxidase (AAO) leading to ABA (Nambara and Marion-Poll, 2005) (**Figure 1**). CCD1 enzymes convert a wide spectrum of carotenoid and apocarotenoid substrates and are also less specific with respect to the targeted double bonds. As shown by *in vitro* studies and functional expression in carotenoid accumulating *Escherichia coli* strains, CCD1 enzymes from different plant species produce the volatiles 6-methyl-5-hepten-2-one (C_8_), geranial (C_10_), and a series of C_13_ cyclohexenones including α- and β-ionone. CCD1 activity leads also to different dialdehyde products, such as rosafluene-dialdehyde that arises simultaneously with the corresponding C_13_-ionone(s) upon the cleavage of C_40_ carotenoids or apo-10’-carotenoids (Vogel et al., 2008; Ilg et al., 2009; Ilg et al., 2014) (**Figure 1**). There are two types of CCD4 enzymes. The first type mediates the cleavage of bicyclic all-*trans*-carotenoids, e.g., all-*trans*-β-carotene, at the C9, C10 or C9’, C10’ double bond leading to apo-10’-carotenoids (C_27_), e.g., β-apo-10’-carotenal, and the corresponding C_13_ cyclohexenone product, e.g., β-ionone (Bruno et al., 2015; Bruno et al., 2016) (**Figure 1**). The second type of CCD4 enzymes forms the *Citrus* pigment citraurin (3-OH-β-apo-8’-carotenoid; C_30_), by cleaving the C7, C8 or C7’, C8’ double bond in hydroxylated bicyclic carotenoids (Rodrigo et al., 2013). CCD7 and CCD8 are strigolactone (SL) biosynthesis enzymes that act sequentially in converting 9-*cis*-β-carotene produced by the carotene isomerase DWARF27 (D27) into the SL precursor carlactone that is the substrate of cytochrome P450 enzymes from the 711 clade, such as the rice carlactone oxidase (OsCO), that form 4-deoxyorobanchol (Alder et al., 2012; Abe et al., 2014; Bruno et al., 2014; Zhang et al., 2014; Bruno and Al-Babili, 2016; Bruno et al., 2017; Abuauf et al., 2018) (**Figure 1**). In addition, CCD7 is also involved in the formation of C_13_ cyclohexenones (Floss et al., 2008b; Walter et al., 2015). ZASs constitute a recently identified CCD subfamily (Wang et al., 2019). A study of the enzymatic activity of a rice ZAS shows that this enzyme converts 3-OH-all-*trans*- β-apo-10’-carotenal (apo-10’-zeaxanthinal) into zaxinone (3-OH-all-*trans*-apo-13-carotenone), a regulatory metabolite required for normal rice growth (Wang et al., 2019).

Besides their role as color and volatile attractants in plant–animal communications (Nisar et al., 2015), apocarotenoids are emerging as key regulators of plant–microbe interactions, in particular of the arbuscular mycorrhizal (AM) symbiosis. AM fungi (AMF) form a widespread root symbiotic association that provides several benefits to the host plants, by improving the mineral nutrition and the tolerance to biotic and abiotic stresses. Furthermore, AMF colonization has an impact on plant developmental processes that determine root architecture, flowering time, fruit and seed formation, and quality (Ruíz-Lozano et al., 2012; Zouari et al., 2014; Fiorilli et al., 2018). The key feature of AM symbiosis is nutrients exchange, in which AMF provide the plant with minerals, mainly phosphorus (P) and nitrogen (N), and receive, in turn, carbohydrates and lipids (Wang et al., 2017 and references within). The AM interaction starts with a chemical dialogue based on plant and fungal diffusible molecules, which triggers the development of fungal adhesion structures on root epidermis. These structures, called hyphopodia, enable the fungus to enter the host root tissues where it spreads *via* intercellular and/or intracellular routes. In the inner cortical layers, fungal hyphae penetrate cortical cells and divide dichotomously, which results in the formation of arbuscules, highly branched structures that are assumed to mediate nutrient exchange (Lanfranco et al., 2018a).

The establishment of the AM symbiosis triggers a cellular, molecular, and metabolic reprogramming of the host plant. Many phytohormones are indeed modulated during the AM colonization and may have themselves a role in regulating the establishment and function of the AM symbiosis (Pozo et al., 2015; Chialva et al., 2018; Liao et al., 2018; Müller and Harrison, 2019). The role of carotenoid metabolism in the AM symbiosis process is not restricted to providing the known plant hormones ABA and SLs. Indeed, several lines of evidence suggest the involvement of other carotenoid-derived metabolites including the recently identified zaxinone (Akiyama et al., 2005; Floss et al., 2008a; Floss et al., 2008b; Walter et al., 2010; Wang et al., 2018; Wang et al., 2019).

In this review we describe the involvement of SLs, ABA, blumenols (C_13_), mycorradicins (C_14_), and zaxinone in AM symbiosis and depict their functional significance during different stages of the AMF colonization process.

### Strigolactones

**Figure 2:**
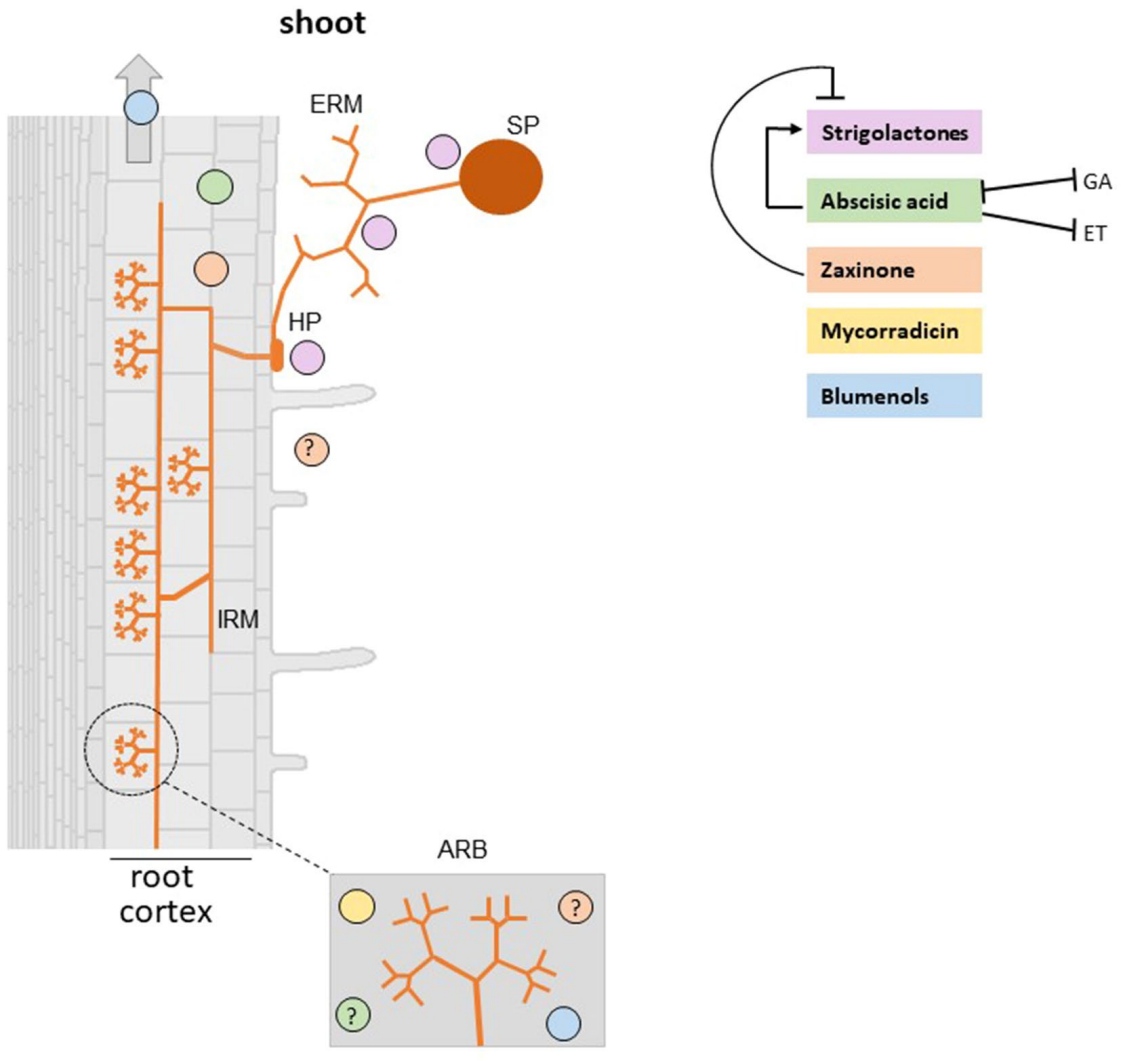
Carotenoid derived-hormones and apocarotenoid signaling molecules involved in the establishment of AM symbiosis. Plant roots release SLs which stimulate AMF spore germination, hyphal branching, hyphopodia formation, and metabolism that in the end promote root colonization. Abscisic acid (ABA) deficient mutants show a reduction of AMF colonization and arbuscule formation and functionality (Herrera-Medina et al., 2007; Martín-Rodríguez et al., 2011). However, it is unclear whether ABA effect on the AM symbiosis is mediated by a cross-talk with SLs, ethylene, and gibberellins. Mycorradicins and blumenols are accumulated in arbuscule-containing cells (Walter et al., 2010). Moreover, Wang et al. (2018) demonstrated that blumenols accumulate also in shoots of several mycorrhizal plants and proposed them as foliar markers for a rapid screening of functional AMF associations. Recent findings showed that zaxinone is produced in mycorrhizal roots and a rice zaxinone defective mutant displays lower AM colonization levels (Wang et al., 2019). SP, spore; HP, hyphopodium; IRM, intraradical mycelium; ERM, extraradical mycelium; ARB, arbuscule-containing cells. Note that the specific localization of ABA and zaxinone is not known (indicated with a question mark). Positive and negative effects are illustrated by arrows and blunt-ended bars, respectively.

Natural SLs are carotenoid-derived compounds characterized by the presence of a methylbutenolide ring (D-ring) linked by an enol ether bridge in (*R*)-configuration to a structurally variable second moiety (Al-Babili and Bouwmeester, 2015). So far, approximately 30 SLs have been isolated from the root exudates of different plant species (Yoneyama et al., 2018). Depending on the structure of the second moiety, natural SLs are classified as canonical SLs that contain a tricyclic lactone (ABC-ring) and non-canonical SLs that have other structures instead (**Figure 1**). Canonical SLs are further divided based on the stereochemistry of the B/C junction into orobanchol- and strigol-like SLs (Al-Babili and Bouwmeester, 2015; Jia et al., 2018). SLs are involved in different developmental processes, including shoot branching, secondary growth, and the establishment of root system architecture (Waters et al., 2017), as well as in plant’s response to biotic and abiotic stress factors (Ha et al., 2014; Decker et al., 2017). In addition, plants release SLs into the soil where they were originally discovered as seed germination stimulants of root parasitic weeds (Xie and Yoneyama, 2010) and later identified as hyphal branching factor for AMF (Akiyama et al., 2005) (**Figure 2**). Since then, SLs have become the best known molecules in early plant–AMF interaction (Lanfranco et al., 2018a; Lanfranco et al., 2018b) and have been shown to be involved in the communication with further beneficial microorganisms, such as rhizobia and in the interaction with detrimental organisms (Marzec, 2016; López-Ráez et al., 2017).

In addition to inducing hyphal branching, SLs trigger a range of responses in AMF, which include spore germination, hyphal elongation, and hyphopodia formation (Lanfranco et al., 2018b and references therein). However, it is still unclear how SLs are perceived by AMF and how they influence AMF. Nevertheless, it has been shown that SLs boost the AMF ATP production and mitochondrial division, activate the expression of mitochondrial and effector genes (Besserer et al., 2006, Besserer et al., 2008; Tsuzuki et al., 2016; Salvioli et al., 2016), and promote the release of chitin oligomers (Genre et al., 2013) that are perceived by the plant partner (Sun et al., 2015). The specific role of SLs during the colonization process within plant roots is still ambiguous; however, SLs deficient mutants show normal arbuscule morphology but lower colonization levels.

It has been shown that carlactonoic acid (CLA), the carlactone oxidation product formed by the *Arabidopsis thaliana* MAX1 (**Figure 1**) and its orthologous in other species, and its derivative methyl carlactonoate (MeCLA) have moderate activity in inducing AMF hyphal branching (Mori et al., 2016). Similarly, lotuslactone, a non-canonical SL that has been recently characterized as a *Lotus japonicus* SL, is a moderate inducer of hyphal branching of AMF (Xie et al., 2019). C_20_ non-canonical SLs, such as heliolactone (sunflower) and zealactone (maize), are also weak inducers of hyphal branching (Xie et al., 2019). However, it is worth to note that hyphal branching of *Gigaspora* species, which is used as a biological assay to determine SL effect, often shows high experimental variability and that only SL-induced hyphal elongation could be confirmed in another AMF species, such as *Rhizophagus irregularis* (Tsuzuki et al., 2016). Therefore, the development of a more reliable assay is desirable for a better comprehension of the biological activity of canonical and non-canonical SLs on AMF.

Taken together, SLs act as positive regulators of the AM symbiosis; they are essential to achieve the full extent of mycorrhization and, probably, more relevant during the early stage of interaction by activating the fungal metabolism and enhancing its ability to colonize roots. Further investigations are needed to clarify the molecular evolution and the biological role of canonical and non-canonical SLs as communication molecules in the rhizosphere.

### Abscisic Acid

The best-studied plant apocarotenoid is the plant hormone ABA (C_15_), which is a key player in plant response to abiotic stress (Peleg and Blumwald, 2011), regulation of plant growth, and development and promotion of pathogen defence responses (Ton et al., 2009; Ma et al., 2018). Strong evidence has emerged from different host plants for a direct role of ABA in mycorrhizal root colonization. *Solanum lycopersicum* (tomato) ABA defective mutants show a reduction of AMF colonization and arbuscule formation and functionality, which may be partially dependent on an increase of ethylene (Herrera-Medina et al., 2007; Martín-Rodríguez et al., 2011). In *Medicago truncatula*, it has been found that ABA promotes AM colonization, but only at low concentration, and that this positive effect is mediated by the protein phosphatase 2A (PP2A) that is activated during the AM symbiosis and upon ABA treatment (Charpentier et al., 2014). More recently, it was reported that *Solanum tuberosum* (potato) plants pre-treated with ABA show higher colonization and arbuscule level (Mercy et al., 2017), suggesting that ABA creates a favorable metabolic environment, possibly during the early stage of mycorrhizal formation. It is worth to note that endogenous ABA levels increase in mycorrhizal roots (Ludwig-Müller, 2010) (**Figure 2**) and that a correlation between ABA and SLs levels was observed (López-Ráez et al., 2010). Both hormones are important for the AM symbiosis and seem to be regulated by each other (López-Ráez et al., 2010; Visentin et al., 2016): this cross-talk may also influence the outcome of the symbiosis. It is also worth to note that antagonistic interactions between ABA and other hormones involved in the AM symbiosis, such as ethylene (Martín-Rodríguez et al., 2011) and gibberellins (GA), have also been demonstrated (Floss et al., 2013; Martín-Rodríguez et al., 2016). In particular, it has been proposed that ABA could regulate AM development by inhibiting ethylene production (Martín-Rodríguez et al., 2011) and contribute in particular to arbuscule formation by modifying bioactive GA levels (Martín-Rodríguez et al., 2016).

### Blumenols (C_13_) and Mycorradicins (C_14_)

Accumulation of specific classes of apocarotenoids, such as mycorradicins and blumenols, is strongly associated with the establishment and maintenance of AMF colonization and can be considered a signature of AM symbiosis (Walter et al., 2007; Hill et al., 2018; Wang et al., 2018). These AMF-induced apocarotenoids can be divided based on their structure in two types: (i) colorless C_13_ cyclohexenone derivatives, such as blumenols, and (ii) yellow C_14_ polyene derivatives, called mycorradicins (Walter et al., 2010; Hill et al., 2018). Mycorradicin and its derivatives are mycorrhizal specific-apocarotenoid mixtures, which are detected thanks to a yellow or yellowish pigmentation of roots (Klingner et al., 1995; Walter et al., 2000; Walter et al., 2007; Walter et al., 2010). Mycorradicin is stored as globules in root chromoplasts and its accumulation leads to changes in root plastid morphology (Scannerini and Bonfante-Fasolo, 1977). Although Fester et al. (2002) identified very low mycorradicin concentrations also in non mycorrhizal roots of some species, its accumulation seems to be specific for the AM symbiosis and not for other symbiotic (such as ectomycorrhizas and nodules) or pathogenic interactions, or for the growth under abiotic stress conditions (Maier et al., 1997; Walter et al., 2010).

In addition to mycorradicins, C_13_ cyclohexenone derivatives, called blumenols, are also accumulated in roots after AMF inoculation (Klingner et al., 1995; Maier et al., 1995; Walter et al., 2000; Strack and Fester, 2006). Blumenols are classified into three major types: blumenol A, blumenol B, and blumenol C (**Figure 1**). However, studies have reported that only the content of blumenol C glycosides is increasing during mycorrhizal colonization. Recently, Wang et al. (2018) found a group of blumenols accumulating in roots and shoots of mycorrhizal plants from different species, i.e., tomato, barley, and potato. Abundance of the five blumenol C-glycosides (11-hydroxyblumenol C-9-O-Glc, 11-carboxyblumenol C-9-O-Glc, 11-hydroxyblumenol C-9-O-Glc-Glc, blumenol C-9-O-Glc-Glc, and blumenol C-9-O-Glc) showed a high correlation with AMF colonization rate, as shown by determining the transcript levels of well-known mycorrhization marker genes. It would be very interesting to know more about the biological function of these compounds that may be responsible for or contribute to the systemic effects (defence, attraction or signaling) exerted by the AM symbiosis on the epigeous portions of mycorrhizal plants. Experiments with exogenous treatments and genetic approaches using mutant lines with reduced or increased accumulation of blumenols will be instrumental to clarify the role of these compounds. In any case, blumenols are foliar markers that extend the possibilities of detecting AM symbiosis and can be used for high-throughput screening for functional AMF-associations (Wang et al., 2018).

Several lines of experimental evidence suggest that C_13_ and C_14_ apocarotenoids originate from a common C_40_ carotenoid precursor through a sequential two-steps cleavage: the current model proposes that a C_40_ carotenoid is cleaved by a CCD enzyme (CCD7 or possibly CCD4), leading to a C_27_ apocarotenoid and C_13_ cyclohexenone; then, the C_27_ apocarotenoid is subsequently converted by CCD1 into rosafluene-dialdehyde (C_14_), the precursor of mycorradicin, and C_13_ cyclohexenone (Floss et al., 2008b; Walter et al., 2010; Hou et al., 2016) (**Figure 1**). The knockdown of *M. truncatula MtDXS2* gene, encoding a 1-Deoxy-D-xylulose 5-phosphate synthase that catalyzes the first step in the plastid isoprenoid biosynthesis, resulted in equal strong reductions of both C_13_ and C_14_ accumulation, which was mirrored by a reduction of the mycorrhizal functionality during later stages of the symbiosis (Floss et al., 2008a). In addition, C_13_ and C_14_ apocarotenoids seem to be strictly linked: both accumulate locally in arbuscules-containing cells (**Figure 2**) where their assumed biosynthetic enzymes are present (Walter et al., 2010). In contrast, *M. truncatula CCD1* knockdown lines displayed a strong reduction in the content of C_14_ mycorradicin derivatives while the C_13_ cyclohexenone level was only moderately affected, indicating that other enzyme(s) are also involved in C_13_ biosynthesis. CCD7, which provides the 9-*cis*-configured C_27_ intermediate in SL biosynthesis, is also a candidate enzyme for synthesizing the C_27_ precursor for the CCD8-mediated formation of the C_18_-ketone β-apo-13-carotenone (Alder et al., 2008) and for the CCD1-catalyzed and AM-induced C_13_ and C_14_ apocarotenoids in mycorrhizal roots (Floss et al., 2008b; Hou et al., 2016). However, it should be mentioned here that CCD7 is a stereospecific enzyme that solely cleaves 9-*cis*-configured carotenoids and forms accordingly configured C_27_-apocarotenoids (Bruno et al., 2014). Therefore, the involvement of CCD7 in the latter two metabolic processes implies a *cis* to *trans* isomerization of the formed C_27_-apocarotenoids, which makes them suitable for being converted into β-apo-13-carotenone and C_13_ and C_14_ mycorrhizal apocarotenoids.

Interestingly, a reduced C_14_ mycorradicin content alone does not hamper the establishment of the AM symbiosis (Floss et al., 2008b), suggesting that C_13_ cyclohexenone derivatives may be more important for a successful AM symbiosis. Indeed, by comparing the distribution of developmental stages of arbuscules in mycorrhizal roots of *M. truncatula DXS* knockdown lines (where both C_13_ and mycorradicin were strongly reduced) and *CCD1* silenced lines (where C_13_ moderately decreased while mycorradicin was strongly reduced), it was found that *DXS* lines displayed a higher ratio between degenerating and dead arbusculues *versus* mature arbuscules. These results provide a hint for a potential function of C_13_ apocarotenoids (or other isoprenoids/apocarotenoids) in arbuscule turnover, and ascribe to mycorradicin a minor contribution in AM establishment and functioning.

### Zaxinone

Zaxinone was recently identified as an important growth-regulating apocarotenoid metabolite in rice. The enzyme responsible for its biosynthesis, ZAS, represents an overlooked sixth CCD subfamily common across the plant kingdom (Wang et al., 2019). ZAS shows the same *in vitro* enzymatic activity of CCD8 with respect to the cleavage of all-*trans*-C_27_ apocarotenoids at position C_13_-C_14_ (Alder et al., 2008; Alder et al., 2012; Wang et al., 2018). Indeed, zaxinone corresponds to a hydroxylated form of the CCD8 product β-apo-13-carotenone (Alder et al., 2008; **Figure 1**).

A rice loss-of-function *zas* mutant shows a lower root zaxinone content, a severely retarded root and shoot growth and higher SL levels. Exogenous application of zaxinone not only rescued the mutant root defects but also promoted root growth in wild-type plants and reduced SL biosynthesis and exudation under low phosphate supply, pointing to a crucial role of zaxinone in rice development and growth. Despite a higher level of SLs, the rice *Oszas* mutant displayed a lower level of AM colonization compared to wild-type plants, although arbuscule morphology was unaltered. This result demonstrates that the cross-talk between zaxinone and SLs during mycorrhization is complex and shows that our understanding of the role of both apocarotenoids in this process is quite limited. So far, *OsZAS* involvement in the AM symbiosis has been only partially characterized: gene expression analyses of rice mycorrhizal roots revealed that *OsZAS* was induced especially during early (7 day post inoculation, dpi) and, to some extent, during late (35 dpi) stages of mycorrhizal colonization (Fiorilli et al., 2015; Wang et al., 2019). However, zaxinone content turned out to increase only during the early phase of the AM interaction, likely before fungal penetration inside the root. This discrepancy might be due to post-transcriptional regulation events of *OsZAS* gene expression and/or a fine balance between zaxinone synthesis and degradation. Another important clue that highlights the role of *OsZAS* in the AM symbiosis is that ZAS orthologues are absent in genomes of non-AMF host species, such as *A. thaliana* (Wang et al., 2019). Further studies are needed to clarify the precise role of zaxinone in the AM symbiosis and to answer the question whether its effect is direct or mediated by additional factors, i.e. alterations of the level of SLs and/or other hormones. The latter question arises from the finding that zaxinone reduces SLs content by acting as a negative regulator of the transcript level of SLs biosynthetic genes. This inhibition may not require the F-box protein D3 (Wang et al., 2019), which is known to be necessary for the SL-dependent negative feedback regulation of SL biosynthesis (Zhao et al., 2015). However, the root growth promoting effect of exogenously applied zaxinone likely requires functional SLs biosynthesis, as it was not observed in SLs deficient mutants (Wang et al., 2019).

Interestingly, a carotenoid compound (D’orenone), with a chemical structure similar to zaxinone, was recently shown to affect ectomycorrhizal formation possibly by modulating auxin metabolism in both partners (Wagner et al., 2016). It can be speculated that zaxinone and D’orenone may have functional similarities in regulating plant development or the interaction with microorganisms. Understanding the biology of zaxinone will provide new insights into plant development and AM symbiosis. Moreover, zaxinone, through its capability to control SLs biosynthesis, has a large application potential as a tool to combat infestations by root parasitic weeds such as *Striga* (Wang et al., 2019), whose weeds require host-released SLs as a germination signal (Al-Babili and Bouwmeester, 2015), which cause enormous crop yield losses in warm and temperate zones (Parker, 2012).

## Conclusions and Perspectives

The carotenoid biosynthesis pathway is an important source of known and postulated hormones and signaling molecules. Some of these carotenoid-derived regulatory metabolites have been recruited for the communication between plants and AMF and as regulators of the process leading to the establishment of a functional AM symbiosis. However, our knowledge on how these metabolites are affecting the symbiosis is still limited. Indeed, the mechanism of action has been unraveled only for SLs during the early stage of the AMF-plant interaction. Further investigations are needed to clarify the precise biological function of ABA, blumenols (C_13_), and mycorradicins (C_14_)-derivatives and the recently identified zaxinone in this process. In addition, the relationships between all these molecules, which originate from the same metabolic pathway, and their interaction with other hormones known to be involved in the AM symbiosis are largely elusive. For this purpose, the characterization of genes encoding CCDs and other carotenoid-modifying enzymes and of their products will remain instrumental for AM symbiosis research and related agricultural application.

## Author Contributions

LL and SA-B proposed the concept. VF and JW organized and drafted the manuscript. PB, LL, and SA-B contributed to the editing of the manuscript. LL and SA-B supervised the work. All authors read and approved the manuscript.

## Funding

This work was supported by baseline funding and Competitive Research Grant (CRG2017) given to SA-B and from King Abdullah University of Science and Technology.

## Conflict of Interest

The authors declare that the research was conducted in the absence of any commercial or financial relationships that could be construed as a potential conflict of interest.
